# Multi-Omics Analysis of Gene and Protein Candidates Possibly Related to Tetrodotoxin Accumulation in the Skin of *Takifugu flavidus*

**DOI:** 10.3390/md19110639

**Published:** 2021-11-15

**Authors:** Huimin Feng, Kun Qiao, Chunchun Wang, Bei Chen, Min Xu, Hua Hao, Zhen Huang, Zhiyu Liu, Qin Wang

**Affiliations:** 1 School of Life Science, Xiamen University, Xiamen 361005, China; fenghuimin@stu.xmu.edu.cn; 2 Fisheries Research Institute of Fujian, Key Laboratory of Cultivation and High-Value Utili-zation of Marine Organisms in Fujian Province, Xiamen 361013, China; qiaokun@xmu.edu.cn (K.Q.); chenbeifjfri@foxmail.com (B.C.); xumin@jmu.edu.cn (M.X.); 3 Fujian Key Laboratory of Special Marine Bio-Resources Sustainable Utilization, College of Life Sciences, Fujian Normal University, Fuzhou 350108, China; qsx20190968@student.fjnu.edu.cn (C.W.); zhuang@fjnu.edu.cn (Z.H.); 4 College of Food and Biological Engineering, Jimei University, Xiamen 361021, China; 5 State Key Laboratory of Marine Environmental Science, College of Oceanography and Environmental Science, Xiamen University, Xiamen 361005, China; hhao@xmu.edu.cn

**Keywords:** *Takifugu* *flavidus*, tetrodotoxin, transcriptome, proteome, accumulate, skin

## Abstract

Pufferfish is increasingly regarded by many as a delicacy. However, the tetrodotoxin (TTX) that accumulates in its body can be lethal upon consumption by humans. TTX is known to mainly accumulate in pufferfish skin, but the accumulation mechanisms are poorly understood. In this study, we aimed to explore the possible mechanism of TTX accumulation in the skin of the pufferfish *Takifugu flavidus* following treatment with TTX. Through liquid chromatography-tandem mass spectrometry (LC-MS/MS) analysis, we detected 37.3% of toxin accumulated in the skin at the end of the rearing period (168 h). Transcriptome and proteome analyses revealed the mechanism and pathways of TTX accumulation in the skin of *T. flavidus* in detail. Gene ontology and the Kyoto Encyclopedia of Genes and Genomes analyses strongly suggest that cardiac muscle contraction and adrenergic signaling in cardiomyocyte pathways play an important role in TTX accumulation. Moreover, some upregulated and downregulated genes, which were determined via RNA-Seq, were verified with qPCR analysis. This study is the first to use multi-omics profiling data to identify novel regulatory network mechanisms of TTX accumulation in the skin of pufferfish.

## 1. Introduction

Pufferfish, also called blowfish, refers to any of approximately 90 species of the family Tetraodontidae. It is common knowledge that pufferfish inflate themselves with air or water into a spherical shape. Puffers are primarily found in the sea and, in some instances, in brackish water or freshwater [1]. Pufferfish are sometimes used as food and have long been regarded as one of the most delicious fish in Japan. However, many pufferfish possess a potent neurotoxin, tetrodotoxin (TTX), which is especially concentrated in the internal organs. In marine pufferfish species, the liver and ovary are highly toxic in general, whereas in brackish water and freshwater species, toxicity is higher in the skin.

TTX was first discovered in 1909 in the ovaries of globefish [2]. TTX is a heterocyclic compound consisting of a guanidinium moiety connected to an oxygenated backbone that possesses a 2,4-dioxaadamantane structure with six hydroxyl groups [3,4,5]. Twenty-five naturally occurring analogs of TTX have been detected [6]; the structures of TTX and its analogs are shown in Table 1. TTX has good water solubility and thermal stability, and its toxicity is not attenuated by cooking, but is rather increased by it [7]. As a sodium channel blocker, TTX can bind to the sodium channels of muscle and nerve tissues, causing poisoning in humans and most animals. However, a wide variety of marine and terrestrial animals are now known to contain TTX, and animals with TTX in their body may be resistant to TTX toxicity [8]. Many researchers have found that in these animals, the aromatic amino acid chain in the p-loop region of domain I in the sodium channels is replaced by a non-aromatic amino acid, which prevents the sodium channels in these species from being blocked [9]. Therefore, TTX is also used to classify voltage-gated sodium channels (VGSCs) as TTX-sensitive or TTX-resistant channels according to their sensitivity to this toxin [10]. TTX-sensitive VGSCs include the Na_v_1.1, Na_v_1.2, Na_v_1.3, Na_v_1.4, Na_v_1.6, and Na_v_1.7 subtypes, and TTX-resistant VGSCs include the Na_v_1.5, Na_v_1.8, and Na_v_1.9 subtypes [11].

TTX is one of the most potent and oldest known neurotoxins. Its accumulation in pufferfish is a complex biological process, the mechanism of which is largely unknown. Many studies have focused on uncovering the specific mechanism; even though these studies have discovered that the amino acid residues of the p-loop regions in domains I and IV of the voltage-gated sodium channels have mutations [9], the TTX transfer and accumulation profiles in pufferfish are still not fully understood. Some studies exploring these mechanisms have shown that when non-toxic cultured specimens of *Takifugu rubripes* or *Takifugu niphobles* are reared with a TTX-containing diet, toxins are efficiently accumulated in their livers and skin, where they are retained for a long period even after the toxic diet is stopped. Many recent studies have used high through-put profiling technologies to identify the related pattern of tetrodotoxin accumulation in pufferfish [12,13], but few have untangled the critical regulatory mechanism between the differentially expressed proteins and mRNAs during TTX transfer and accumulation in the skin [14,15]. Transcriptomics and proteomics have been used to identify important molecular mechanisms involved in various biological processes [16]. Thus, a combined transcriptomic and proteomic approach is needed to clarify the mechanism of TTX transfer and accumulation.

*T. flavidus* belongs to the family Tetraodontidae. The skin, ovary, and liver of the wild-type *T. flavidus* are toxic [17]. Researchers have found that pufferfish can secrete tetrodotoxin through the skin, thus protecting juvenile fish from predators [18]. However, non-toxic specimens of puffer can be cultured via rearing with a TTX-free diet [19]. Our previous research found that the non-toxic specimens of *T. flavidus* could also be cultured using the same method. The breeding technology for *T. flavidus* is now mature, as the demand for pufferfish is increasing year by year and has a high market value. To use *T. flavidus* as an edible pufferfish species, we should quickly figure out the transfer and accumulation mechanism of TTX to ensure safe consumption.

In this study, tetrodotoxin was used to gavage *T. flavidus,* and the toxin content of different tissues after gavage was examined. We performed transcriptome and proteome analyses on the skin of *T. flavidus* at seven days after oral gavage administration of TTX to investigate the effect of TTX accumulation into the skin on gene expression and to identify the genes and proteins possibly related to TTX accumulation in the skin. Through the analyses of differentially expressed genes and enriched proteins, genes and proteins that play a role in the accumulation of TTX and resistance to TTX can be identified. This study would answer questions beyond just the transcriptomics and proteomics changes that may help drive TTX accumulation in the skin. Furthermore, this study will contribute to a better understanding of how the skin responds to TTX toxicity and help determine whether the pufferfish defends against predator attacks through the TTX of the skin.

## 2. Results

### 2.1. Accumulation of TTX in the Skin

**Figure 1:**
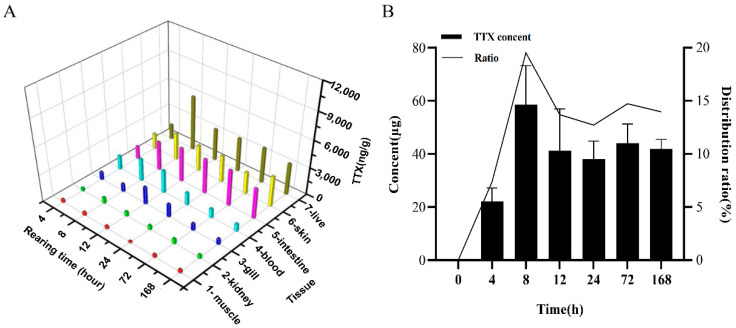
Toxin accumulation. (**A**) Toxin transfer and accumulation at different time points. (**B**) TTX concentration in the skin at different time points and the TTX accumulation ratio (percentage [%] of accumulated TTX in skin tissue [µg/mass of sample] to the remaining dose [µg/kg]) of the body.

TTX was orally administered via gavage to non-toxic cultured specimens of the pufferfish *T. flavidus* to investigate the toxin transfer and accumulation profiles in the skin. TTX was not detected in all the TTX-free groups. However, in all TTX treatment groups, TTX was detected at high levels in the skin and liver, and at low levels in the other tissues (Figure 1A). TTX concentration in the skin showed an upward trend within 8 h (2956.7 µg/kg), fluctuated between 8 and 72 h, and reached a maximum at 168 h (3386.7 µg/kg). Liquid chromatography with tandem mass spectrometry (LC-MS/MS) analysis revealed TTX content in the skin at different times after toxin administration, as well as the TTX accumulation ratio, that is, the ratio (%) of TTX accumulated in skin tissues (µg/mass of sample) to the remaining dose (µg/kg). The results are shown in Figure 1B. TTX content showed an upward trend, and the TTX accumulation ratio reached 30.1% within 8 h. TTX content decreased at 8‒24 h. After 24 h, the amount of TTX accumulated in the skin hardly changed. However, the TTX accumulation ratio changed slightly at 8‒24 h, indicating that the trends of TTX transfer and accumulation in the skin were the same as those in the whole body. At 168 h, the TTX accumulation ratio reached a maximum (37.3%). These findings imply that when TTX enrichment, transportation, and metabolism are in a dynamic equilibrium state, the skin is the main organ of TTX accumulation. 

### 2.2. Transcriptomic Landscape in the Skin Tissue of T. flavidus and Identification of Differentially Expressed Genes (DEGs)

**Figure 2:**
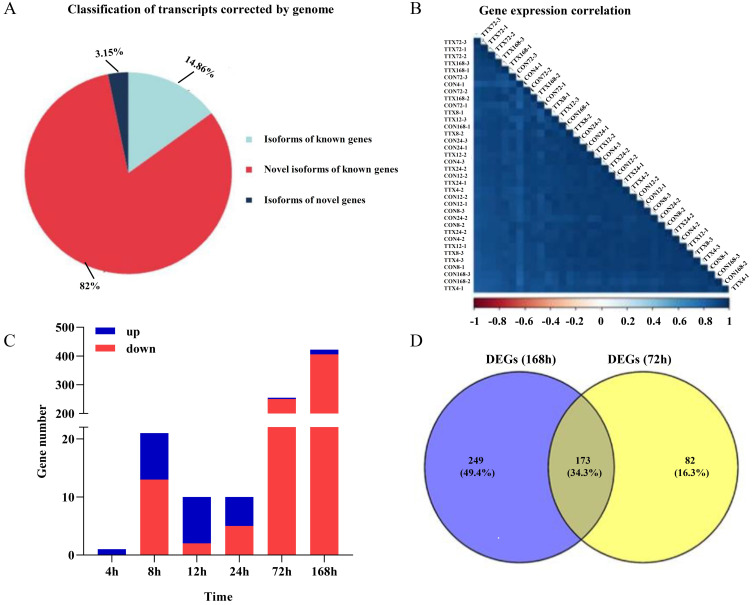
RNA sequencing results of *T. flavidus* skin to elucidate the TTX accumulation process. (**A**) Full-length transcript classification. (**B**) Gene expression correlation analysis. (**C**) Up- and downregulated DEGs of the TTX-gavage groups vs. TTX-free groups at 4 h, 8 h, 12 h, 24 h, 72 h, and 168 h. (**D**) Venn diagram of DEGs of the TTX-gavage groups vs. TTX-free groups at 72 h and 168 h.

Approximately 28.7 G polymerase reads and 27.88 G subreads were obtained from transcriptome samples (Tables S1 and S2). Next, we obtained 466,891 CCS reads from the subreads and divided the sequences into full-length sequences, yielding 392,939 FLNC reads. The hierarchical n × log(n) algorithm was subsequently used to cluster and polish the full-length sequences to obtain a high-quality consensus sequence of 31,073 reads (minimum length, 50 bp; maximum length, 15,000 bp) (Table S3). Illumina sequencing obtained raw base 230.56 G, and clean base 221.17 G. The mapping efficiency between the reads and the reference genome of each sample was between 88.3% and 92.1%, and the details of the sequencing and mapping results are provided in Table S4. Next, the high-quality, Illumina-seq data were subjected to sequencing error correction on Iso-seq reads (Table S5). Consensus sequences were aligned and polished according to the reference genome using GMAP, resulting in 91.7% of the consensus sequence (Table S6). The consensus sequence that could be compared to the genome contained 16,397 isoforms, including 2436 isoforms of known genes, 13,445 novel isoforms of known genes, and 516 isoforms of novel genes (Figure 2A). Functional annotations were performed on the unmapped isoforms, structurally annotated isoforms, and novel genes in seven databases (NR, NT, PFAM, KOG/the Clusters of Orthologous Groups (COG), Swiss-Prot, KEGG, and GO) (Figure S1A–C).

Pearson’s correlation analysis indicated good repeatability between the samples (Figure 2B). As shown in Figure 2C, a total of 719 genes were differentially expressed in the CTTX-treated groups compared to the control groups, with *p*_-_value < 0.05. Of these, 43 genes were upregulated, and 676 genes were downregulated. Most of the DEGs were screened at 72 h and 168 h. This phenomenon proved that gene expression in *T. flavidus* was significantly affected at the end of the rearing period, suggesting that the physiological function of *T. flavidus* might be significantly affected by TTX treatment. Using a Venn diagram, we identified a total of 173 identical DEGs in the TTX-treated groups vs. TTX-free groups at 72 h and 168 h (Figure 2D). 

**Figure 3:**
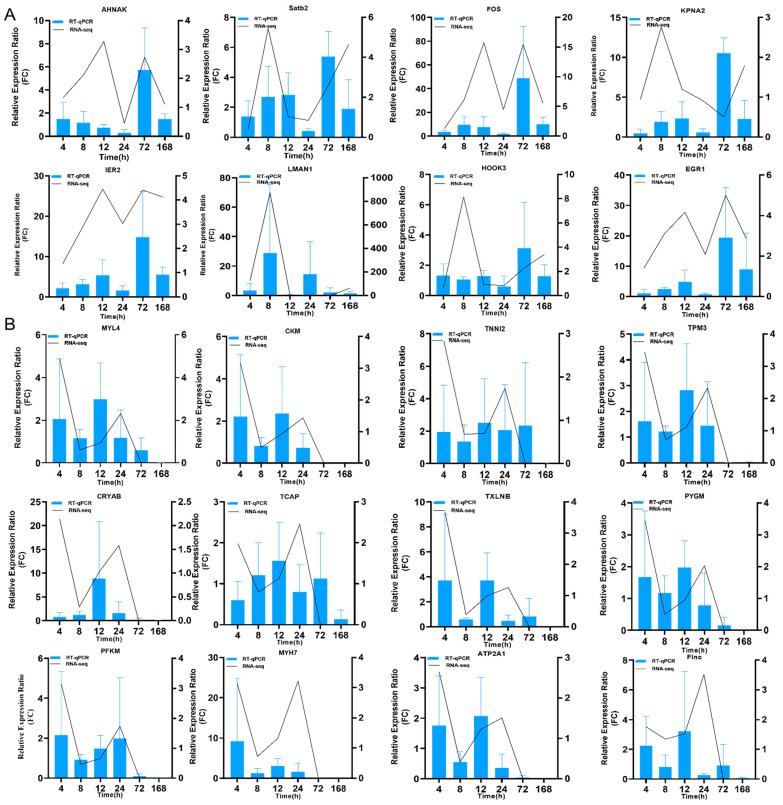
qPCR verification of DEGs from RNA-seq results. (**A**) RT-qPCR verification of the selected upregulated DEGs at different time points. (**B**) RT-qPCR verification of the selected downregulated DEGs at different time points.

To verify the reliability of transcriptome data, 20 DEGs, which included 8 upregulated genes and 12 downregulated genes, were selected for expression level analysis using RT-qPCR. Regarding the expression of these DEGs at different times, the results of qPCR analyses were not completely consistent with those of RNA sequencing (RNA-Seq) analysis, including those of 8 upregulated DEGs (Figure 3A) and 12 downregulated DEGs (Figure 3B). However, the expression of these DEGs at 72 and 168 h, according to qPCR analysis, was consistent with the results of RNA-Seq analysis (Figure S2A,B). The correlations of changes in the expression of 16 DEGs between the RNA-Seq and qPCR results were analyzed by the linear-fitting method, with R^2^ of 0.8649 and 0.7964 in the 72-h and 168-h groups, respectively (Figure S2C,D). The results indicated that the transcriptional changes observed by qPCR were consistent with the results obtained by RNA-Seq, thereby confirming the RNA-Seq data.

### 2.3. Functional Enrichment of the DEGs

**Figure 4:**
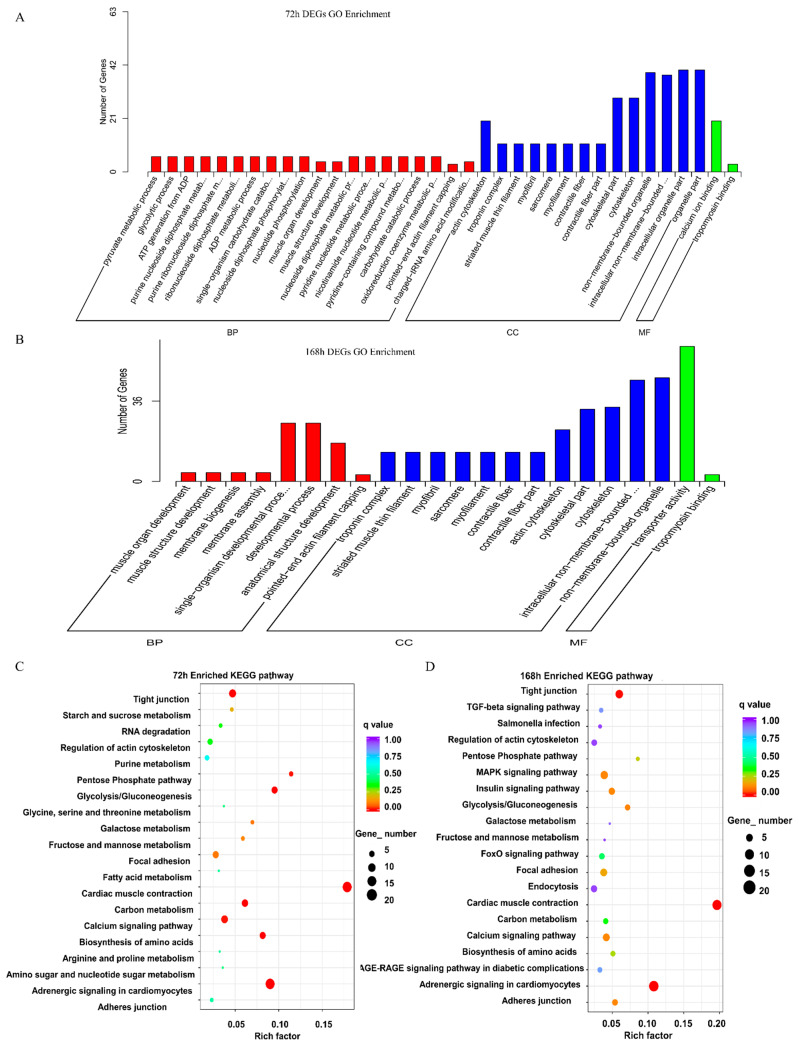
GO and KEGG analysis of DEGs. (**A**,**B**) Gene Ontology (GO) enrichment analysis of the DEGs in the 72-h (168 h) group according to the cellular component, molecular function, and biological process categories. (**C**,**D**) Kyoto Encyclopedia of Genes and Genomes (KEGG) pathway classification enrichment analysis of the DEGs in the 72-h (168 h) group. The y-axis and x-axis indicate the KEGG pathway and Rich factor of the differentially expressed genes of different pathways based on transcriptome sequencing, respectively.

To further evaluate the functions of the unigenes, functional enrichment analysis was performed on the up- and downregulated DEGs to detect significantly enriched Gene Ontology (GO) and Kyoto Encyclopedia of Genes and Genomes (KEGG) pathways using the GOseq program. In the 72-h group, 255 DEGs were classified into three major categories: biological processes (BP), cellular components (CC), and molecular function (MF). A total of 39 significantly enriched GO terms were enriched in the three major functional categories (*p* < 0.01). The main GO terms are shown in Figure 4A. The seven major terms of BP were pyruvate metabolic process, glycolytic process, ATP generation from ADP, purine nucleoside diphosphate metabolic process, purine ribonucleoside diphosphate metabolic process, ribonucleoside diphosphate metabolic process, and ADP metabolic process. The eight major terms of CC were actin cytoskeleton, troponin complex, striated muscle thin filament, myofibril, sarcomere, myofilament, contractile fiber, and contractile fiber. The two major MF terms were calcium ion binding and tropomyosin binding.

In the 168-h group, 422 DEGs were classified into BP, CC, and MF in 17 GO terms (BP and CC, *p*-value < 0.01; MF, *p*-value < 0.05). The main GO terms are shown in Figure 4B. The five significantly enriched GO terms of BP were muscle organ development, muscle structure development, membrane biogenesis, membrane assembly, and the single-organism developmental process. The seven significantly enriched GO terms of CC were troponin complex, striated muscle thin filament, myofibril, sarcomere, myofilament, contractile fiber, and contractile fiber part. The two major significantly enriched GO terms of MF were transporter activity and tropomyosin binding. 

To further study the biochemical metabolic pathways and other pathways related to TTX accumulation in the skin, KEGG analysis was conducted using the KEGG pathway database. There were 57 KEGG pathways in the 72-h group, and the top 20 pathways are shown in Figure 4C. The most representative pathways were cardiac muscle contraction (20 unigenes), adrenergic signaling in cardiomyocytes (20 unigenes), glycolysis/gluconeogenesis (8 unigenes), biosynthesis of amino acids (8 unigenes), carbon metabolism (9 unigenes), tight junction (11 unigenes), pentose phosphate pathway (4 unigenes), and calcium signaling pathway (10 unigenes). In the 168-h group, there were 78 KEGG pathways, and the top 20 pathways are shown in Figure 4D. The most represented pathways were cardiac muscle contraction (22 unigenes), adrenergic signaling in cardiomyocytes (24 unigenes), tight junction (14 unigenes), glycolysis/gluconeogenesis (6 unigenes), insulin signaling pathway (9 unigenes), MAPK signaling pathway (13 unigenes), calcium signaling pathway (11 unigenes), and adherens junction (7 unigenes). These results indicated that cardiac muscle contraction, adrenergic signaling in cardiomyocytes, glycolysis/gluconeogenesis, tight junctions, and calcium signaling pathways play important roles in TTX enrichment. Venn diagram presenting the total KEGG pathway enrichment of DEGs between the 72-h and 168-h groups shows a total of 49 KEGG pathways enriched in both groups (data not shown). These pathways are thus considered to be related to TTX accumulation in the skin.

### 2.4. Results of Proteomic Study

**Figure 5:**
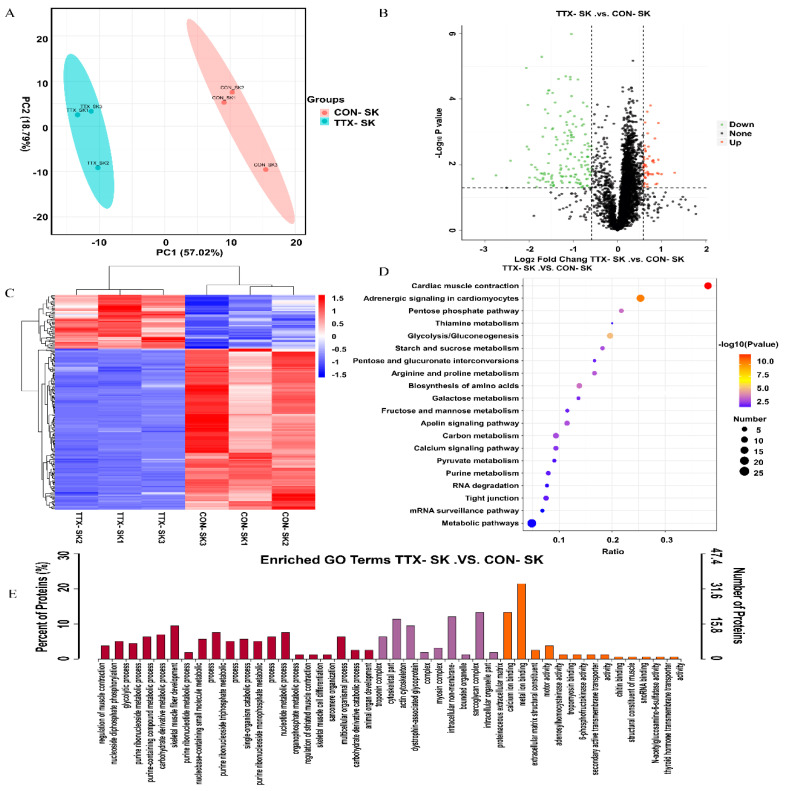
Analysis of proteomic results. (**A**) PCA analysis. (**B**) Volcano plot of differentially expressed proteins. (**C**) Heat map with hierarchical clustering of three differentially expressed proteins. Hierarchical clustering analysis demonstrated a clear divergence in the proteomes of the TTX-168 h and CON-168 h groups, with only minimal differences between the biological replicates. (**D**) KEGG pathway classification enrichment analysis of DEPs. (**E**) GO enrichment analysis of the DEPs. The y-axis and x-axis indicate the percentage of annotated proteins and GO terms, respectively.

Since the 168-h group had the largest number of DEGs and enriched pathways, proteomic detection and analysis were performed on this group. The characteristics of *T. flavidus* included in this study differed between the TTX_168 h and CON_168 h groups. Figure S3A shows the proteomic study procedure. Principal component analysis of sample replicates from the TTX_168 h and Con_168 h groups (n = 3 per group) revealed high variability (Figure 5A). The distribution of unique peptide numbers shows that more reliable proteins have been identified (Figure S3B) and that the distribution of the peptide length agreed with the properties of tryptic peptides (Figure S3C). In total, we identified 4368 proteins, including 4358 quantifiable proteins (Table S7). The functions and domains of the identified proteins were annotated using the GO, KEGG, COG, and IPR databases (Figure S3D). We compared the proteomes of the 168-h group. Proteins with *p*-value < 0.05 and FC > 1.5 were considered significantly differentially expressed; thus, 200 differentially expressed proteins (DEPs) were identified (Table S8). Among these DEPs, 150 were downregulated and 50 were upregulated (Figure 5B). Cluster analysis of the 200 DEPs identified in the TTX_168 h and CON_168 h groups showed different results (Figure 5C). The DEPs were subjected to functional enrichment analysis to detect significantly enriched GO and KEGG pathways. The enriched GO terms were also classified by MF, CC, and BP terms, as shown in Figure 5E. Among them, regulation of muscle contraction, nucleoside diphosphate phosphorylation, glycolytic process, purine ribonucleoside metabolic process, purine-containing compound metabolic process, carbohydrate derivative, metabolic process, skeletal muscle fiber development, purine ribonucleotide metabolic process, troponin complex, cytoskeletal part, actin cytoskeleton, dystrophin-associated glycoprotein complex, myosin complex, calcium ion binding, metal ion binding, and extracellular matrix structural constituents were the significantly enriched GO terms.

To further study the biochemical metabolic pathways and signal transduction pathways related to TTX accumulation, KEGG analysis was conducted using the KEGG pathway database. The 200 DEGs were annotated to 46 pathways in KEGG analysis (Figure 5D). The pathways included cardiac muscle contraction, adrenergic signaling in cardiomyocytes and glycolysis/gluconeogenesis. DEPs for subcellular localization (Figure S3E) included 18.9% nuclear protein, 17.5% cytoplasmic protein, 16.8% plasma membrane protein, 14.0% extracellular protein, and 10.5% cytoskeleton protein.

### 2.5. Summary of Transcriptomic and Proteomic Analyses

**Figure 6:**
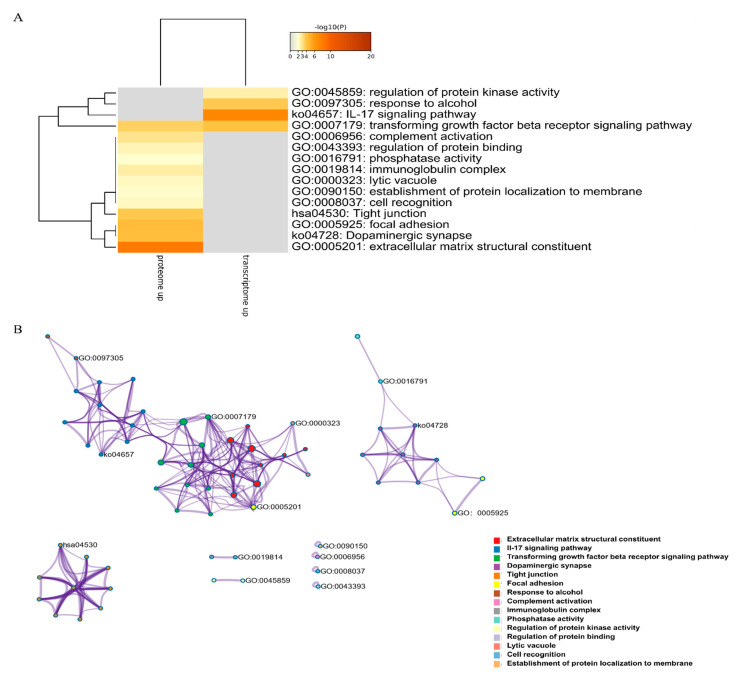

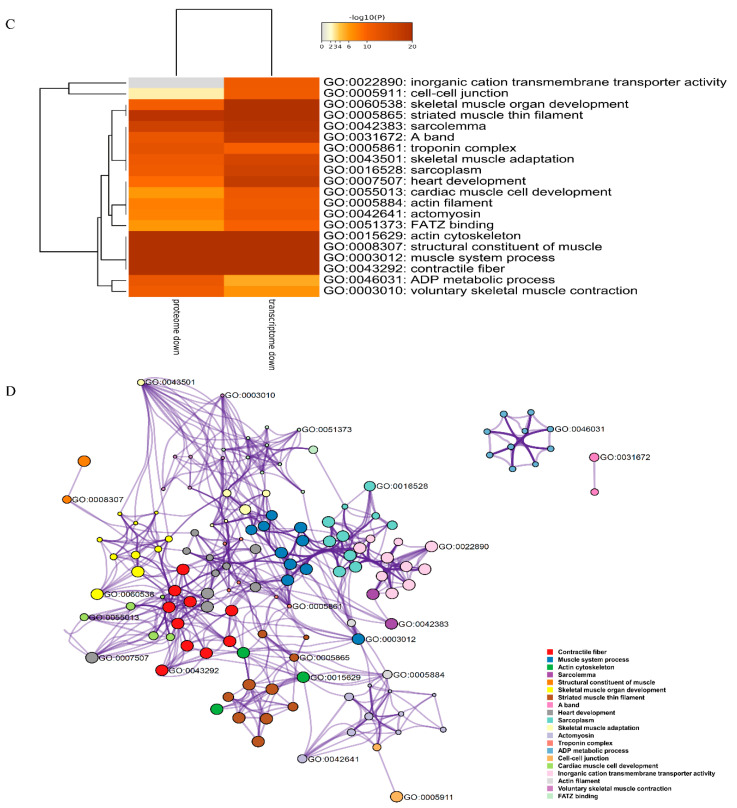

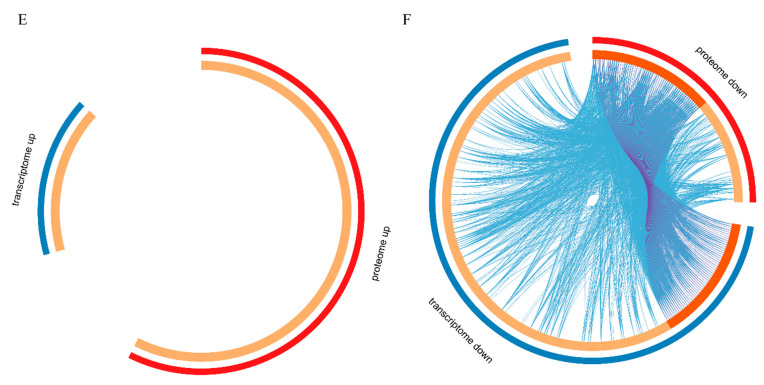
Enrichment analysis. (**A**) Heatmap of enriched terms across upregulated (downregulated) DEGs (colored by *p*-values). (**B**) Upregulated (downregulated) DEG network of enriched terms, colored by cluster ID, where nodes that share the same cluster ID are typically close to each other. (**C**) Heatmap of enriched terms across upregulated (downregulated) DEPs (colored by *p*-values). (**D**) Upregulated (downregulated) DEP network of enriched terms, colored by cluster ID, where nodes that share the same cluster ID are typically close to each other. (**E**,**F**) Overlap between gene lists, including the shared terms, where blue curves link genes that belong to the same enriched ontology term. The inner circle represents gene lists, where hits are arranged along the arc. Genes belonging to multiple hit lists are colored in dark orange, and genes unique to a list are shown in light orange.

DEGs and DEPs were subjected to pathway enrichment analysis. Metascape can integrate different omics data, such as transcriptomics and proteomics data, followed by pathway and process enrichment analyses. We obtained the top 20 clusters of DEPs and DEGs as well as their representative enriched GO terms and KEGG pathways, and then chose the most significant terms of upregulated DEGs and DEPs and downregulated DEGs and DEPs (those with the lowest *p*-value) as a representative of each cluster (Figure 6A,C). The upregulated DEGs and DEPs were enriched in regulation of protein kinase activity, response to alcohol, IL-17 signaling pathway, transforming growth factor-beta receptor signaling pathway, and complement activation. The downregulated DEGs and DEPs were enriched in inorganic cation transmembrane transporter activity, cell-cell junction, skeletal muscle organ development, striated muscle thin filament, and sarcolemma. The results strongly suggested the importance of these pathways in TTX accumulation in pufferfish skin. To further elucidate the relationships between these terms, a subset of enriched terms was selected and rendered as a network plot, where terms with a similarity >0.3 were connected by edges, with each node representing an enriched term and colored first by its cluster ID (Figure 6B,D). The overlap between DEGs and DEPs is shown in Figure 6E,F. Upregulated DEGs and DEPs did not share any enriched ontology terms, but downregulated DEGs and DEPs shared many enriched ontology terms.

## 3. Discussion

This study showed that TTX was transferred and accumulated in different tissues of *T. flavidus*. Studies have found that different species of pufferfish have different TTX transfer and accumulation profiles, with the liver and skin being the main sites of TTX accumulation [20]. In marine species of *T. rubripes*, the liver and ovaries have the highest toxicity, followed by the intestines. However, in marine species of *T. flavidus*, the liver, ovary, skin, and intestines have the highest toxicity [21]. Previous studies have investigated TTX transfer and accumulation profiles in non-toxic cultured specimens of the pufferfish *T. rubripes* by intramuscularly administering crude extract of the toxic pufferfish ovary (cTTX). The results suggested that 15–23% of the administered toxin was transferred and retained in the liver for up to 24 h, and 89% of the remaining toxin was transferred and accumulated in the skin at 168 h [22]. Intramuscularly-administered TTX in pufferfish is rapidly transferred to the skin, liver, and other tissues, with a high concentration of TTX in blood plasma; then, TTX is transferred to the skin via the bloodstream within 72‒168 h [23]. The epidermal layer of the skin comprises basal cells and sacciform cells. Immunohistological studies found that TTX appeared brown in both basal cells and sacciform cells of the pufferfish *Tetraodon steindachneri* and *Tetraodon nigroviridis* at 168 h after administration [24]. Taken together, these findings show that TTX is transferred mainly to the liver and skin of *T. flavidus* following short-term administration, and that TTX can accumulate in the skin of *T. flavidus* by transfer via the bloodstream from other tissues. The results of this study indicate that the liver is the main organ for TTX metabolism and excretion and that the skin may play an important role in TTX accumulation. The results show that the accumulation and transfer of TTX have species and tissue specificity.

In phagocytosis, foreign materials are digested by hydrolytic enzymes in lysosomes, and these enzymes require highly acidic environments. Research has shown that acidic conditions are conducive to the transport and accumulation of TTX. The SLC26 family plays a vital role in mediating the exchange of Cl^−^/HCO_3_^−^ in epithelial tissues, and SLC26A6 can mediate the transport of Cl^−^/HCO_3_^−^ as well as other anions, including Cl^−^/HCOO^−^, Cl^−^/C_2_O_4_^2−^, Cl^−^/NO^3−^, SO_4_^2−^/ C_2_O_4_^2−^, and Cl^−^/OH^−^ [25]. Additionally, Cl^−^ influx and HCO_3_^−^ efflux mediated by SLC26A6 may be beneficial for intracellular acidification. Our results showed that an acidic environment was created in the skin to accumulate TTX transferred from other tissues within 4 h of administration.

As a transcriptional cofactor, special AT-rich binding protein-2 affects gene expression by regulating chromatin architecture. The expression of interstitial markers, such as vimentin, fibronectin, *N*-cadherin, and α-smooth muscle actin, was increased. The dynein-activating adaptor Hook3 is required to activate dynein/dynactin motility, and experiments on the dynein/dynactin/Hook3 and KIF1C complexes have indicated that KIF1C can transport dynein/dynactin toward the microtubule plus ends and that dynein/dynactin can transport KIF1C toward the microtubule minus ends [26]. Considering that the expression of these genes was increased during the rapid accumulation of TTX in pufferfish skin, the above processes may be related to the transfer and accumulation of TTX in pufferfish skin.

c-Fos and c-Jun are activator protein (AP-1) transcription factors, which is a group of immediate-early response genes (IEG) that respond rapidly to various stimuli [27]. c-Fos or c-Jun levels are commonly used for evaluating neuronal activities caused by inflammatory or neuropathic pain [28,29]. Knockdown or dominant-negative inhibition of c-Fos or c-Jun in sensory neurons reduces neuropathic pain [30]. There is a strong correlation between the activation of IEG and the induction of transporter synthesis, and IEG activation may contribute to TTX transfer. The expression of Fos proto-oncogene (FOS) and Jun proto-oncogene (JUN) may change to adapt to environments with high concentrations of TTX. However, importin a3 (Kpna4) perturbs c-Fos nuclear import in sensory neurons, reduces sensitivity to noxious stimuli, and exerts analgesic effects specifically during the maintenance phase of neuropathic pain [31]. 

The present study showed a large decrease in muscle fiber expression both at the gene and protein levels, including those of myosin light chain 2, myosin light chain 4 (MYL4), myosin heavy chain 7 (MYH7), titin (TTN), tropomyosin 2, and tropomyosin 3. The decrease in muscle fiber gene expression may be related to TTX accumulation. The network we generated was condensed and interconnected with genes of many different functions, which indicated that the TTX transfer and accumulation process may occur in cascades. This suggests that all the included genes can affect each other and play a role in TTX transfer and accumulation in the skin through unknown mechanisms. We found that genes, such as Fos, Jun, MYH, MYL, IEG, and TNNI, may be related to TTX transfer and accumulation. 

## 4. Materials and Methods

### 4.1. Pufferfish Specimens

Non-toxic cultured specimens of *T. flavidus* (approximately 12 months old; body weight, 100 ± 30 g; body length, 15 ± 2 cm; *n* = 72) were purchased from a culture farm in Zhangpu, Fujian Province, China.

### 4.2. Toxin Administration Experiments

Twenty milligrams of crude TTX were dissolved in 1 mL of acetate buffer (0.1 M, pH = 4.0) and diluted with 19 mL of phosphate-buffered saline (1 M). The pufferfish specimens were divided into two groups of 36 individuals; one group was administered crude TTX (Taizhou Kangte Biological Engineering Co., Ltd.; Taizhou, China. CTTX group), and the other was administered phosphate-buffered saline/acetate buffer (20:1, *v*/*v*, control group). Each fish was anesthetized with Eugenol cement/ alcohols/H_2_O (1:9:3 × 10^4^, *v*/*v*/*v*), then orally administered 0.5 mL (1 mg/mL) of either crude TTX solution or 0.5 mL phosphate-buffered saline/acetate buffer via gavage and then immediately returned to the tank (total handling time < 30 s for each individual to minimize stress to the fish). Six fish from each group were randomly collected at 4, 8, 12, 24, 72, and 168 h after toxin administration, and toxin quantification was performed as described below.

#### 4.2.1. Preparation of Samples

All specimens were dissected into different anatomic tissues (skin, liver, kidney, muscle, gill, and intestine). Each tissue was washed with PBS, and then 1 g of tissue was mixed and ground with acetic acid (0.05 M)/methanol (1:99, 4.5 mL), ultrasonically extracted in 50 °C water bath for 15 min, centrifuged at 7200× *g* for 5 min, and then the cleared supernatant was transferred to a new centrifuge tube. The above procedure was repeated, and the obtained supernatants were combined and diluted to 25 mL, followed by freezing at −20 °C for 30 min. Next, the pooled supernatant was centrifuged at 7200× *g* for 5 min, diluted 5 times with PBS solution, and then the pH was adjusted to 7‒8 using 1 mol/L sodium hydroxide. Using a syringe precoated with sodium heparin, blood was withdrawn from the portal vein of each fish and centrifuged at 2000× *g* for 10 min.

#### 4.2.2. LC-MS/MS

Next, 1 mg of TTX standard was dissolved in formic acid (1 M)/H_2_O (1:999, 2 mL), and the volume was adjusted to 10 mL with methanol, followed by the addition of formic acid (0.1%, *v*/*v*) and acetonitrile (1:1, *v*/*v*) to make a standard intermediate solution of 1 μg/mL. Thereafter, the standard intermediate solution was diluted with a formic acid solution (0.1%, *v*/*v*) and acetonitrile solution (1:1, *v*/*v*), and then the volume was made constant to obtain a standard series of working solutions with a concentration of 1 μg/L–1000 μg/L. All samples were submitted for examination via LC-MS/MS at the Fujian Inspection and Research Institute for Product Quality [26]. LC-MS/MS was performed using formic acid solution (1:999, *v*/*v*) containing 5 mM ammonium acetate buffer and acetonitrile (1:9, *v*/*v*) as a mobile phase at a flow rate of 0.3 mL/min at 40 °C. LC-MS/MS experiments were recorded on a mass spectrometer equipped with an ESI source in the positive-ion mode. ESI was evoked by a spray voltage of +2 kV, and the heated capillary temperature was maintained at 350 °C. Multiple Reaction Monitoring (MRM) mode mass acquisition was used for monitoring TTX fragment ions. Fragment ion with *m*/*z* 312 as selected as precursor ion and *m*/*z* 302 and 162 were selected as production from protonated [M+H] + from TTX. Two transitions were used for qualitative (identification and confirmation purposes) and quantitation of TTX. The dwell time was set at 200 ms per Da and the collision energy at 40 and 26 eV. Argon was used as the target gas. The tissue sample was purified by an immunoaffinity column. The PBS solution sealed in the immunoaffinity column was released at a natural flow rate, transferred into the tissue sample, and passed through the column at a flow rate of 1 drop/s, rinsed with water (10 mL), and eluted with acetic acid (1 M)/methanol solution (2:98, 5 mL), then dried under nitrogen at 45 °C. The sample was dissolved with formic acid (0.1 M)/acetonitrile solution (1:1, 1 mL), subjected to ultrasound for 1 min, then filtered through a syringe filter (0.22 µm). The sample was subjected to LC-MS/MS analysis to obtain the response peak area and the concentration of TTX in the test solution according to the standard curve.

### 4.3. RNA Extraction and Sequencing

RNA was prepared as described by Qiao et al. [32]. Total RNA was extracted from *T. flavidus* skin tissues using TRIzol reagent (Invitrogen, Burlington, ON, Canada). The quality and integrity of RNA samples were determined using a Nanodrop ND1000 (Thermo Fisher Scientific, Wilmington, NC, USA) and Agilent 2100 bioanalyzer (Agilent Technologies,), respectively. Library preparation was performed as described by Li et al. [33]. The Iso-Seq library was prepared according to the Isoform Sequencing protocol (Iso-Seq) [34], and the RNA-seq library was prepared according to NovaSeq 6000 Sequencing protocol [35], then sequencing was performed at Nanjing Novogene Bio Technology Co., Ltd. (Nanjing, China). After Isoform sequencing was completed, the subreads were obtained, and then the circular consensus sequence (CCS) algorithm was used to obtain the CCS. Next, based on the 5′-primer, 3′-primer, and Poly-A, CCSs were classified into full-length non-chimera (FLNC) and non-full-length (nFL) sequences. The FLNCs of the same transcript were clustered using the hierarchical n × log(n) algorithm to obtain a consensus sequence. Finally, the obtained polished consensus sequence was used for subsequent analysis [36]. After Illumina NovaSeq 6000 sequencing was completed, the raw data were obtained, and then reads with adapters, reads containing N (N means the base information cannot be determined), and low-quality reads (Qphred ≤ 20 bases account for 50% of the total read length) were removed to obtain clean data for subsequent analysis [37].

### 4.4. Reads Mapping, Gene Structure Analysis, and Gene Transcript Functional Annotation

Additional nucleotide errors in consensus reads were corrected using Illumina RNA-seq data. Consensus reads were aligned to the reference genome using a genomic mapping and alignment program (GMAP) [38]. Reference genome and gene model annotation files were downloaded directly from the genome website (http://fishomics.gooalgene.com/#/download, accessed on 23 September 2021). We selected Hisat2 (v2.1.0) as the mapping tool. Gene structure analysis was performed using the Transcriptome Analysis Pipeline from Isoform Sequencing (TAPIS) [39]. Gene transcript functions were annotated based on the following databases: NR (NCBI non-redundant protein sequences), NT (NCBI non-redundant nucleotide sequences), Pfam (Protein family), KOG/COG, Swiss-Prot (a manually annotated and reviewed protein sequence database), KO (KEGG Ortholog database), and GO. We used the BLAST software and set the e-value to “1 × 10^−10^” in NT database analysis [40]. For NR, KOG, Swiss-Prot, and KEGG database analyses, we used the Diamond BLASTX software and set the e-value to “1 × 10^−10^.” The Hmmscan software was used for Pfam database analysis [41].

### 4.5. Identification of DEGs

Differential gene expression analysis of the treatment groups (three biological replicates per condition) was performed using the DESeq R package (1.18.0). The resulting *p*-values (*p*-value < 0.05) were adjusted using the Benjamini and Hochberg approach for controlling the false discovery rate (FDR). Corrected *p*-values of 0.005 and log_2_(fold change) of 1 were set as the threshold for significant differential expression.

### 4.6. Validation of Transcriptome Analyses Using Quantitative PCR

To validate the DEG transcriptome analysis results, total RNA samples from the treatment groups (CTTX and BPS) were subjected to qPCR analysis. cDNA was synthesized from 1000 ng of total RNA from each sample using a Primescript RT reagent kit (TaKaRa, Dalian, China). The qPCR reactions contained 1 μL of cDNA, 1.5 μL of forward and reverse primers, 10 μL of SYBR mixture, and 7.5 μL of nuclease-free water. RT-qPCR was performed using the SYBR Premix Ex Taq kit (TaKaRa). The primers used in this study were designed using the free online software Primer-BLAST from NCBI. All primers were synthesized commercially by Sangon Biotech (Shanghai, China). All primers are shown in Table S9. The relative expression of the genes was quantified using the 2^−ΔΔCT^ method with the efficiency correction normalized to EF-1α.

### 4.7. Total Protein Extraction and Tandem Mass Tag (TMT) Labeling of Peptides

Samples were ground individually in liquid nitrogen and lysed with 2 mL of lysis buffer, which contains 100 mM NH_4_HCO_3_ (pH 8), 8 M urea, and 0.2% (*v*/*v*) SDS, followed by 5 min of ultrasonication on ice, then centrifuged at 12,000× *g* for 15 min at 4 °C. At first, the supernatants of samples were reduced through a water bath (56 °C) with 10 mM dithiothreitol (DTT) for 1 h, and subsequently alkylated in the dark (1 h, 25 °C). The samples were then completely mixed with the precooled acetone (1:4, *v*/*v*) by vortexing and incubated at −20 °C (2–3 h), then centrifuged and collected these precipitates. After washing twice with cold acetone, these pellets were dissolved in a dissolution buffer containing 0.1 M triethylammonium bicarbonate (TEAB, pH 8.5) and 6 M urea [42]. Next, 120 μg of each protein sample was dissolved 100 μL dissolution buffer, followed by the addition of trypsin (1.5 μg) and TEAB buffer (500 μL), then mixed and digested at 37 °C for 4 h. Subsequently, 1.5 μg trypsin and calcium chloride were added, and the sample was digested overnight. Formic acid was mixed with the digested sample (pH < 3) and centrifuged at 12,000× *g* for 5 min at 25 °C. The supernatant was slowly loaded onto the C18 desalting column, washed three times, and eluted, then the eluents were lyophilized. The sample was mixed with TEAB buffers and acetonitrile dissolved TMT labeling reagent (100:41, *v*/*v*) shaking for 2 h at 25 °C. The reaction was stopped by the addition of 8% (*v*/*v*) ammonium hydroxide (NH_4_OH) [43]. Using Q ExactiveTM HF-X mass spectrometer (Thermo Scientific), Nanospray Flex™ (ESI) ion source (Thermo Scientific), set the ion spray voltage to 2.3 kV, and the ion transfer tube temperature to 320 °C. The mass spectrometer adopts a data-dependent acquisition mode. The scanning range is *m*/*z* 350–1500.

### 4.8. The Identification and Functional Analysis of DEPs

The resulting spectra from each run were searched separately using Proteome Discoverer 2.2 (PD 2.2, Thermo, Carlsbad, NM, USA). A maximum of two mis-cleavage sites was allowed. Peptide spectrum matches (PSMs) with a credibility of more than 99% were included in the analysis. Proteins containing at least one unique peptide were also included. The identified PSMs and proteins were retained and quantified with an FDR of no more than 1.0%. The protein quantification results were statistically analyzed using *t*-tests. Proteins with significantly different levels between the experimental and control groups (*p* < 0.05, |log_2_FC| > 0.58 [fold change, FC]) were defined as DEPs. GO and InterPro (IPR) functional analyses were conducted using the interproscan program against non-redundant protein databases (Pfam, PRINTS, ProDom, SMART, ProSite, and PANTHER) [44], and COG and KEGG were used to analyze the protein family and pathway. DEPs were subjected to volcanic map analysis, cluster heat map analysis, as well as GO, IPR, and KEGG enrichment analyses [45].

### 4.9. Enrichment Analysis

To elucidate the biological roles of the identified DEGs and DEPs, we conducted pathway enrichment analysis using Metascape (https://metascape.org/, accessed on 28 September 2021) tools. By inputting the lists of DEGs and DEPs simultaneously, Metascape can identify commonly enriched and selectively enriched pathways from two levels, which enables a comprehensive assessment of the molecular features of the biological process [46].

### 4.10. Statistical Analysis

The GraphPad Prism 8 software and Origin 2021 software were used for statistical analyses. Significant differences were determined via one-way analysis of variance (ANOVA), followed by Fisher’s least significant difference post-hoc test. Differences with *p* < 0.05 were considered significant.

## 5. Conclusions

After gavage administration of TTX to *T. flavidus*, a large amount of TTX accumulated in the skin in a short time, leading to increased expression of transcription factors (Fos, Jun, and Ier2) and related response stimuli. This suggests that TTX transport and accumulation in the skin may be related to the activation of these transcription factors. In addition, the expression of muscle fiber genes (MYH, MYL, and TNNI) and glycogen metabolism-related genes (PKM, PFKM, and PYGM) decreased, indicating that the massive accumulation of TTX has an impact on the hypertrophic cardiomyopathy pathway and cardiac muscle contraction pathway.

## Figures and Tables

**Table 1:** The structures of TTX and its analogs.

TTX	anhydro-TTX	6-epi-TTX	11-deoxy-TTX
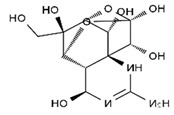	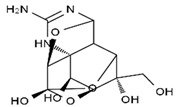	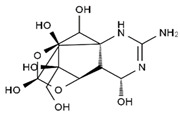	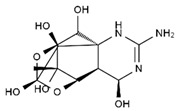
methoxy-TTX	4-ethoxy-TTX	6-dehydroxymentyl-TTX	deoxy-TTX
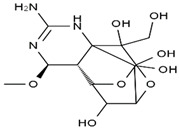	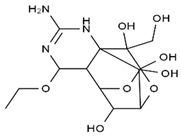	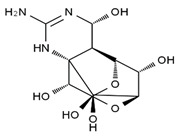	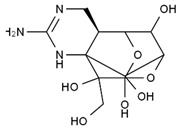
diacetylanhydro-TTX	11-oxo-TTX	4-epi-TTX	4,9-anhydro-TTX
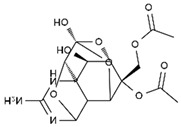	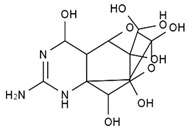	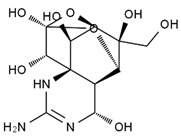	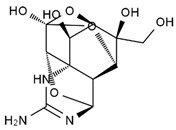
4,9-anhydro-TTX	8,11-dideoxy-TTX	8-deoxy-TTX	11-nor-TTX-6(R)-ol
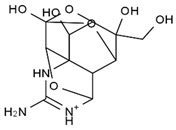	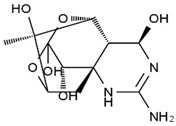	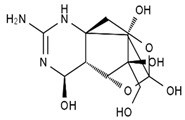	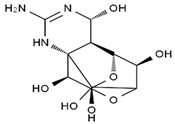
11-nor-TTX-6(S)-ol	11-nor-TTX-6,6-diol	5-deoxy-TTX	6-deoxy-TTX
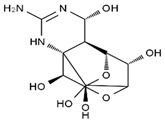	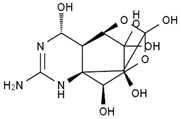	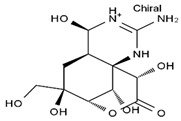	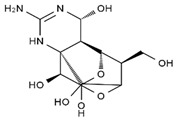
6,11-dideoxy-TTX	chiriquitoxin	tetrodaminotoxin	4-beta-TTX
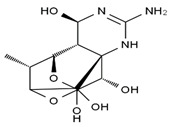	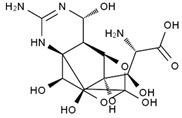	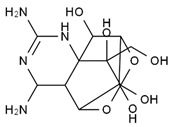	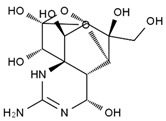
11-carboxymethyl amino-11-deoxy-TTX		N,N′-ethylenediaminedi-TTX	
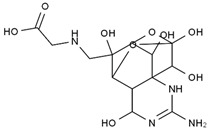		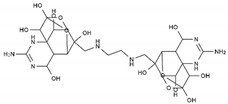	

## Data Availability

The clean reads were deposited in the SRA database of GenBank with the BioProject accession numbers PRJNA766711 and PRJNA766793.

## Supplementary Materials

The following are available online at https://www.mdpi.com/article/10.3390/md19110639/s1, Figure S1: Functional annotation in seven databases (NR, NT, PFAM, KOG/COG, SwissProt, KEGG, GO); Figure S2: qPCR verification of the RNA-seq result of DEGs; Figure S3: Quality control and functional annotation of identified proteins; Table S1: Polymerase read statistics; Table S2: Subread statistics; Table S3: Classification statistics of CCS, FLNC, and consensus; Table S4: Illumina sequence data and mapping efficiency; Table S5: Consensus sequence length statistics before and after transcript correction; Table S6: Polished consensus sequence mapped to the reference genome; Table S7: Number of peptides and proteins identified; Table S8: Number of differentially expressed proteins; Table S9: The primer sequence for differential expression analysis.
