# Supplementary material for: Multi-Omics Analysis of Gene and Protein Candidates Possibly Related to Tetrodotoxin Accumulation in the Skin of Takifugu flavidus
Source: Mar Drugs. 2021 Nov 15;19(11):639. doi: 10.3390/md19110639 (PMC8621849; doi:10.3390/md19110639)
Supplement: Supplementary file 1 [file marinedrugs-19-00639-s001.zip › marinedrugs-1440608-supplementary.pdf]

Table S1

Polymerase read statistics

Polymerase Read Bases (G)	Polymerase Reads	Polymerase Read Length(mean)	Polymerase Read N50
28.7	583625	49169	113836

*Polymerase reads: High-quality sequencing reads produced by a single molecule

Table S2

Subreads statistics

Subread base(G)	Subreads number	Average subreads length	N50
27.88	11234648	2483	2747

*Subreads: Filtered polymerase read

Table S3

Classification statistics of CCS, FLNC and Consensus

Read	number	Min length	Max length	Mean length	N50
CCS	466891	57	15000	2847	3079
FLNC	392939	52	14910	2736	2975
Consensus	31073	372	11772	2770	3051

*CCS: The consensus sequence obtained after self-alignment correction

FLNC: a non-chimeric sequence with 3’, 5’ primers and a poly A tail

Consensus: Sequence after cluster and remove redundancy FLNC

Table S4

Illumina sequence data and mapping efficiency

sample	Raw Base(G)	Clean Base(G)	mapping efficiency
CON_4hS1	7.63	7.33	90.84%
CON_4hS2	6.53	6.23	92.04%
CON_4hS3	6.66	6.41	91.89%
CON_8hS1	6.78	6.47	90.83%
CON_8hS2	6.47	6.24	91.78%
CON_8hS3	6.66	6.39	90.52%
CON_12hS1	6.57	6.33	90.91%
CON_12hS2	6.46	6.19	91.40%
CON_12hS3	6.55	6.22	91.04%
CON_24hS1	6.73	6.44	91.23%
CON_24hS2	6.46	6.08	90.08%
CON_24hS3	6.94	6.64	91.19%
CON_72hS1	7.29	7.02	90.22%
CON_72hS2	6.41	6.18	90.90%
CON_72hS3	6.6	6.31	92.05%
CON_168hS1	7.15	6.89	91.47%

CON_168hS2	7.28	7	91.57%
CON_168hS3	7.28	7.01	91.12%
TTX_4hS1	6.84	6.57	91.56%
TTX_4hS2	7.07	6.67	88.31%
TTX_4hS3	6.83	6.6	91.06%
TTX_8hS1	7.23	6.85	91.88%
TTX_8hS2	6.31	6.03	91.11%
TTX_8hS3	6.49	6.25	91.61%
TTX_12hS1	6.6	6.16	91.50%
TTX_12hS2	8.2	7.99	90.38%
TTX_12hS3	6.28	6.06	91.98%
TTX_24hS1	6.53	6.33	90.42%
TTX_24hS2	7.04	6.8	90.07%
TTX_24hS3	6.27	6.17	90.44%
TTX_72hS1	6.51	6.3	91.38%
TTX_72hS2	6.83	6.52	91.07%
TTX_72hS3	6.67	6.4	91.79%
TTX_168hS1	6.41	6.09	90.84%
TTX_168hS2	6.73	6.49	91.16%
TTX_168hS3	6.62	6.37	91.48%

Table S5

Consensus sequence length statistics before and after transcript correction

Type	Total nucleotides	Total number	Mean length	Min length	Max length	N50	N90
Before correct	86046645	31073	2770	372	11772	3051	1820
After correct	86036010	31073	2769	372	11774	3050	1820

Table S6

Polished consensus sequence map to the reference genome

Total reads	Total mapped	Unmapped	Multiple mapped	Uniquely mapped	Reads map to “+”	Reads map to “-”
31073	29502 (91.73%)	2671 (8.27%)	67 (0.22%)	28435 (91.51%)	14418 (46.4%)	14017 (45.11%)

Table S7

Number of peptides and proteins identified

MS/MS spectrum database search analysis summary				
Total spectra	Matched spectrum	Peptide	Identified proteins	Quantifiable proteins
341245	46001	25204	4368	4358

Table S8

Number of differentially expressed protein

Differentially expressed protein summary		
	regulated type	fold change>1.5
TTX/CON	up-regulated	50
	down-regulated	150

Table S9

The primer sequence of differential expressed genes

Gene Name	Primer Sequence
EF1α	ACTGAGGTGAAGTCTGTGGAAA
	TTTGGTGGGTCGTTCTTGCTGTC
FOS	TGCGACTCTTCTTCCCGTTG
	CTTGATGCTGTCAGGTCGGT
Satb2	CCAGCGCTACAACGTCAAAC
	GCAACGCTGCACATGATTCT
IER2	ATCCTGGCAGTGTCGATCAG
	CGGGCACTTCGCATAACCAT
LMAN1	TGATGGCGAAGAACGCAGAG
	CACAGGCGGGTTTGATCCAT
HOOK3	GGAGGAACTTCGGAAAGCCA
	AGAGTCTCGTTCCGTCCTCA
EGR1	AGGCTCACGTCCCAAAATCA
	TGCGGATGTGAGTGGTAAGG
AHNAK	ATCACTCGCCGCATCACTAC
	CTCCAGACTCATGCCGTAGC
MYL4	ATGTGGAGACGTGATGCGAG
	TGAGCCCCTCCACAAAATCC
CKM	TCCTGACTTGTGCCGGTATG
	GAATCGCCTGAAGACCTCCC
TNNI2	TGCTGACGCCATGTTGAAAG
	CCTCCACATTCTTACGCCAGT
TPM3	CGGGGTCCATTCAGCCTTTT
	TTTGTCCAGCTCGTCCTCAG
MYH7	GGAGGACCGGAAGAATGTCG
	GAGATGAGTGTTGGCCTGCT
CRYAB	AAGGATGGTCCCGGCTTTAC
	CAGCATCGGAGCGGATATGA
TCAP	AGAGCTACAACGCCGAATGG
	AAGTGCCCACTCTGAAGACC
TXLNB	CAGTGACCCAGTTCAGGTCC
	TGCTTCTTAGCGGAGGTGTC
PYGM	CCCTTGTCAGACCACGACAG
	TGTGGGCGAGAGCAAAGTAG

PFKM	ATATGCAACAGGGCGGAACA
	GAGTTGGCAAAGATGCGACC
KPNA2	AGAACCCCTCACCCCCTTTA
	GCACCGTCCGTCAAGTATGA
ATP2A1	CAACTTCTGGCTGCTTGCTG
	GCGACAAACTTCAGCACCTC
Flnc	CCGTTAACTTCCTCGTCCCC
	ATGGTGATTGTGCCGTCCTT

Supplementary Figures

Figure S1

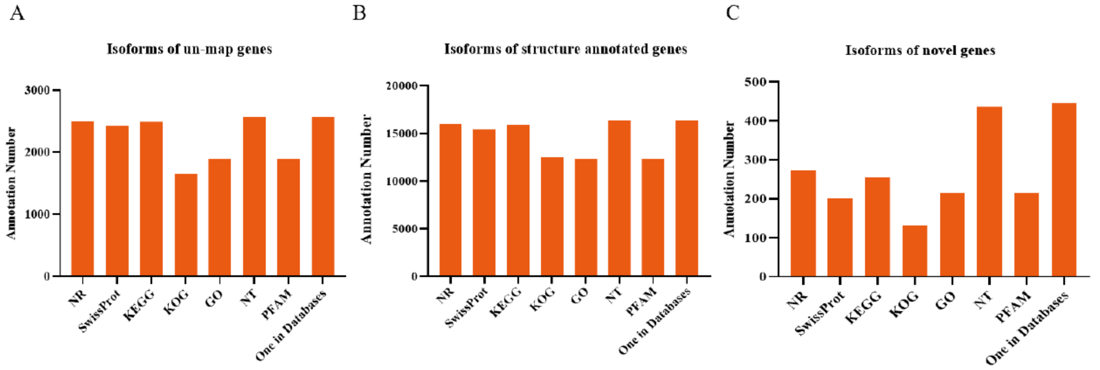

Fig.S1 Functional annotation in seven databases (NR, NT, PFAM, KOG/COG, SwissProt, KEGG, GO). A. unmapped isoforms. B. the structurally annotated isoforms. C. the novel genes.

Figure S2

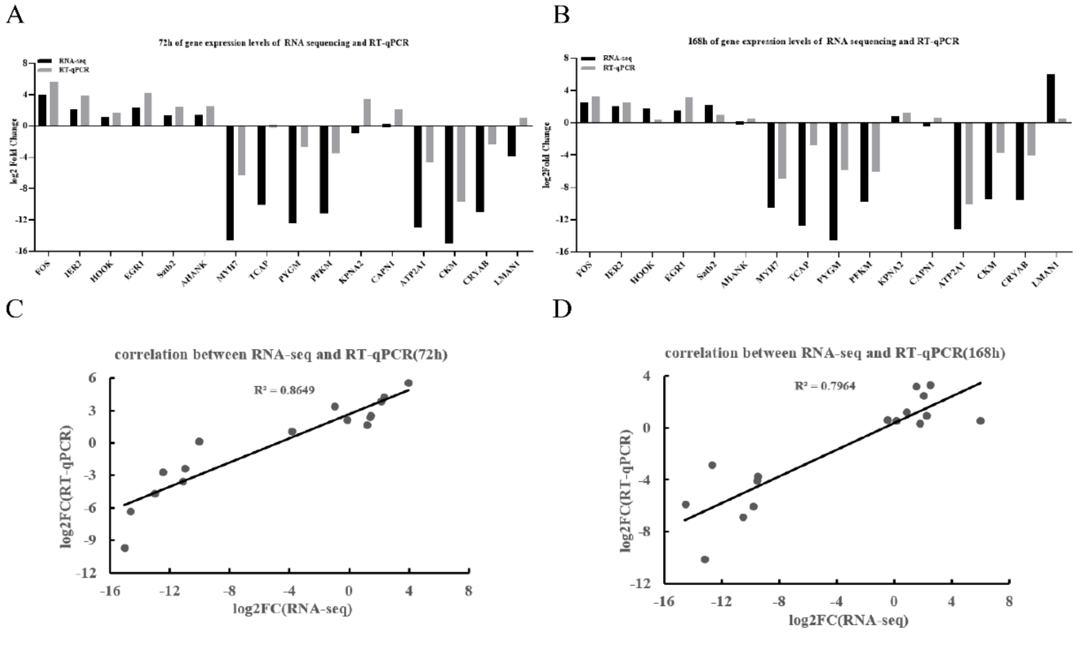

Fig.S2 qPCR verity the RNA-seq result of DEGs. A(B). Comparison of gene expression levels of 16 DEGs of 72h (168h) between RNA sequencing and RT-qPCR analyses. C(D). Correlation analysis between RT-qPCR and RNA-Seq results of 16 DEGs of 72h (168h) indicating a positive correlation (linear-fitting method (R2)).

Figure S3

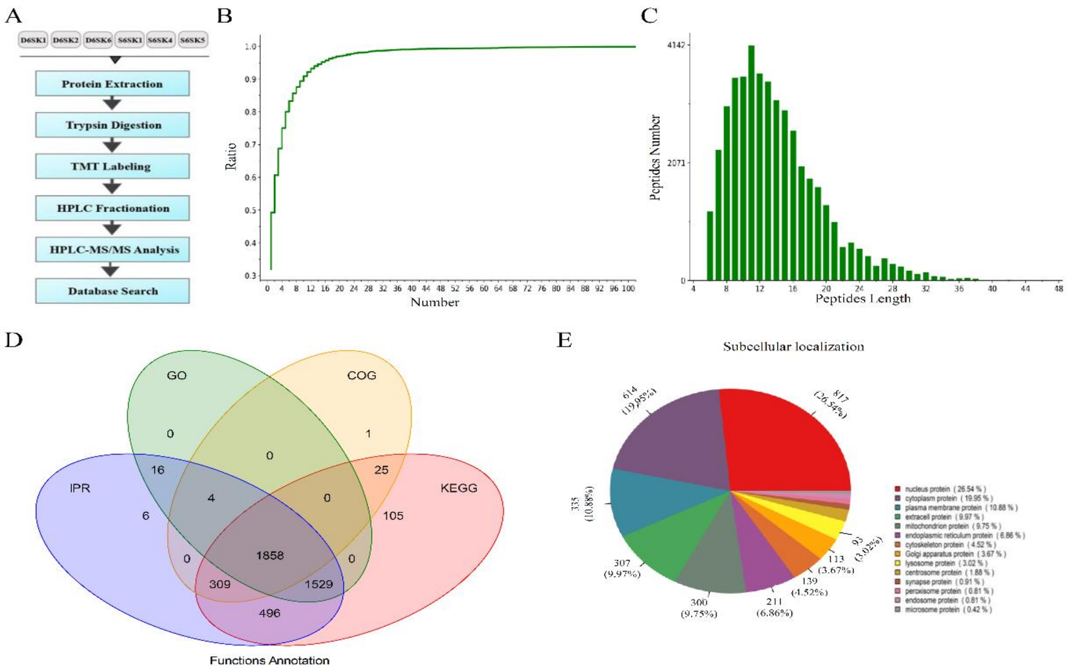

Fig.S3 Quality control and functional annotation of identified proteins. A The procedure of the proteomic study. B. The distribution of Unique peptide number. C. Peptide length distribution identified by quantitative proteomic analysis. D. Functional annotation of the identified proteins. E. Subcellular localization of DEPs.
